# Procedural learning is impaired in dyslexia: Evidence from a meta-analysis of serial reaction time studies^☆^

**DOI:** 10.1016/j.ridd.2013.07.017

**Published:** 2013-10

**Authors:** Jarrad A.G. Lum, Michael T. Ullman, Gina Conti-Ramsden

**Affiliations:** a Deakin University, Melbourne, Australia; b Georgetown University, Washington, DC, United States; c The University of Manchester, United Kingdom

**Keywords:** Dyslexia, Serial reaction time, Procedural learning, Implicit learning, Meta-analysis, Systematic review

## Abstract

A number of studies have investigated procedural learning in dyslexia using serial reaction time (SRT) tasks. Overall, the results have been mixed, with evidence of both impaired and intact learning reported. We undertook a systematic search of studies that examined procedural learning using SRT tasks, and synthesized the data using meta-analysis. A total of 14 studies were identified, representing data from 314 individuals with dyslexia and 317 typically developing control participants. The results indicate that, on average, individuals with dyslexia have worse procedural learning abilities than controls, as indexed by sequence learning on the SRT task. The average weighted standardized mean difference (the effect size) was found to be 0.449 (CI_95_: .204, .693), and was significant (*p* < .001). However, moderate levels of heterogeneity were found between study-level effect sizes. Meta-regression analyses indicated that studies with older participants that used SRT tasks with second order conditional sequences, or with older participants that used sequences that were presented a large number of times, were associated with smaller effect sizes. These associations are discussed with respect to compensatory and delayed memory systems in dyslexia.

## Highlight

• A systematic review and meta-analysis was used to investigate whether procedural learning is impaired in dyslexia.
• The review confirms dyslexia is associated with a procedural learning impairment.
• Differences in study findings may reflect compensatory mechanisms associated with the declarative memory system.

## 1 Introduction

Individuals with developmental dyslexia have significant difficulties with reading despite appropriate educational opportunities and an absence of intellectual impairments or an identifiable disease or disorder that might otherwise account for the problem (American Psychiatric Association, 2000; World Health Organization, 1996). Dyslexia is one of the most common learning impairments, with prevalence estimates from data collected in the United States and other western countries varying from 3% to 7% (Barbiero et al., 2012; Shaywitz, Shaywitz, Fletcher, & Escobar, 1990).

Behavioral investigations have revealed a pattern of deficits in dyslexia beyond reading impairments. Evidence suggests that difficulties with phonological processing may constitute the core impairment in dyslexia, in particular problems with phonological awareness, that is, the ability to identify and manipulate the sound structure of words in a language (Snowling, 2000). Research has also revealed a range of impairments and problems in other domains. This includes impairments in visual processing (Stein & Walsh, 1997), auditory processing (Tallal, 2004), working memory (Gathercole, Alloway, Willis, & Adams, 2006), oral language (McArthur, Hogben, Edwards, Heath, & Mengler, 2000) and motor functioning (Ramus, Pidgeon, & Frith, 2003). However, the relationship between the reading and other co-occurring problems in dyslexia is still unclear. In particular, it remains a subject of ongoing debate which, if any, of the cognitive, language, and/or motor impairments may best account for the reading problems in the disorder (Bishop & Snowling, 2004; Rosen, 2003).

### 1.1 Dyslexia and procedural memory impairments

Similarly, there has been ongoing interest in whether one or more functions of the procedural memory system also contribute or underlie the reading impairments in dyslexia (Nicolson & Fawcett, 1990, 2007; Nicolson, Fawcett, Brookes, & Needle, 2010; Ullman, 2004). This memory system underlies the learning, knowledge, and execution of motor and cognitive skills and habits (Gabrieli, 1998; Packard & Knowlton, 2002; Ullman, 2004). The system underlies a range of types of knowledge, including context-dependent sequential or probabilistically structured information. Learning and knowledge in this system seems to be implicit (not available to conscious awareness), and the learned skills can be processed automatically and rapidly. Learning the skills is relatively slow, with a fair amount of repetition or practice required in order for them to be processed rapidly and automatically. The neural substrates of the procedural memory system are also reasonably well understood, with the basal ganglia, cerebellum, and motor-related areas all playing roles (Kandel, Schwartz, & Jessell, 2012; Packard & Knowlton, 2002; Parent & Hazrati, 1995; Ullman, 2004).

It has been hypothesized that the reading impairments in dyslexia may be at least partly explained by problems with the procedural memory system. Nicolson and Fawcett (2007, 2011) argue that the reading difficulties in dyslexia are, in part, related to parts of the procedural memory system that support language, in particular phonology. Specifically, it is claimed that the reading problems in dyslexia are linked to problems with learning and/or adapting phonological knowledge and automatizing skills necessary to support reading. Nicolson and Fawcett particularly implicate the cerebellum in dyslexia. Ullman (2004) also posits the presence of procedural memory impairments in dyslexia, but suggests that the underlying neural abnormalities may encompass various brain structures underlying procedural memory, including the basal ganglia. Consistent with these views, neural abnormalities have been reported in various structures underlying procedural memory, including the cerebellum (Brambati et al., 2004; Kronbichler et al., 2008), the basal ganglia (Eckert et al., 2005; Pernet, Poline, Demonet, & Rousselet, 2009), and motor areas (Silani et al., 2005). Finally, both Nicolson (Nicolson & Fawcett, 1990, 2007) and Ullman (Ullman, 2004; Ullman & Pullman, 2013) consider that the declarative memory system plays a compensatory role for at least some of the procedural memory deficits in dyslexia.

A key prediction of the proposal that procedural memory impairments are found in and may underlie dyslexia, is that individuals with dyslexia should in fact have worse procedural learning abilities than control individuals with typical reading skills. A number of studies have examined learning in procedural memory in dyslexia, using a variety of paradigms, including artificial grammar learning (Pavlidou, Louise Kelly, & Williams, 2010; Rüsseler, Gerth, & Münte, 2006), alternating serial reaction time task (Howard, Howard, Japikse, & Eden, 2006), as well as the classic serial reaction time (SRT) task first described by Nissen and Bullemer (1987). Indeed, many of these studies have reported procedural learning impairments in the disorder (e.g., Vicari et al., 2005; Vicari, Marotta, Menghini, Molinari, & Petrosini, 2003). However, this finding has not always been replicated (e.g., Bussy et al., 2011; Deroost et al., 2010; Menghini et al., 2010), leaving open the question as to whether procedural learning deficits are indeed found in dyslexia. Moreover, the heterogeneity of findings suggests the possibility that participant level variables (e.g., the age of tested individuals) or methodological factors (e.g., the amount of training in the learning tasks) might help explain the pattern of results.

Qualitative reviews cannot easily synthesize this literature, while accounting for study-specific features such as effect size, sample size, and task related methodological differences. Rather, a rigorous quantitative approach using meta-analytic techniques is more appropriate. In meta-analysis, the results from similar individual studies with similar methodologies are pooled, permitting population parameters to be estimated with greater precision (Borenstein, 2009; Hunter & Schmidt, 1990).

### 1.2 The serial reaction time (SRT) task

This report used meta-analysis and meta-regression to evaluate and synthesize existing evidence and determine whether procedural learning is affected in dyslexia and what factors may influence the observed inconsistencies in the literature. We focused on the SRT task because this task has been the most widely used to examine procedural learning in dyslexia, with fully 14 studies to our knowledge, and indeed, is the best-studied procedural learning task more generally (for a brief review of findings related to the SRT task see Robertson, 2007). Thus, it is now appropriate to summarize this literature using meta-analysis.

In the SRT task, which was initially developed by Nissen and Bullemer (1987), participants are seated in front of a computer display, on which a visual stimulus repeatedly appears in one of four locations. In the implicit version of the task, on which we focus here, the only instructions provided are to press one of several (typically four) buttons that matches the location of a visual stimulus on the screen. For example, if the second of four stimuli in a row light up, the participant must press the second of four buttons in a row as quickly and accurately as possible. The primary dependent variable of interest is reaction times (RTs) that measure how rapidly participants are able to press the response button following presentation of each visual stimulus. The task is typically divided into blocks of stimulus presentations. Within each block there may in the range of 50 to over 100 stimulus presentations, depending on the study. Unknown to participants, in most blocks the visual stimulus follows a predefined sequence that is typically 6–12 items in length. This sequence is repeated multiple times within the ‘sequence blocks’. Following training on the sequence blocks, a ‘random block’ is presented in which the visual stimulus appears randomly.

In neurologically intact children and adults (e.g., Lum, Kidd, Davis, & Conti-Ramsden, 2010; Thomas et al., 2004), RTs decrease (i.e., becomes faster) over the course of training on the sequence blocks, but then increase on the random block. This increase in participants’ RTs (i.e., RTs become slower) that is observed when the visual stimulus begins to appear randomly, is taken to indicate that information about the sequence has been learned. Note that if no information about the sequence had been obtained, RTs should continue to decrease or reach asymptote, presumably as participants become proficient at pressing the response buttons. This latter result is often found in individuals with neurodegenerative diseases or lesions affecting basal ganglia or cerebellum (Knopman & Nissen, 1991; Mayor-Dubois, Maeder, Zesiger, & Roulet-Perez, 2010; Molinari et al., 1997; Pascual-Leone et al., 1993; Siegert, Taylor, Weatherall, & Abernethy, 2006) or who have prefrontal lesions (Beldarrain, Grafman, Pascual-Leone, & Garcia-Monco, 1999; Schmidtke, Manner, Kaufmann, & Schmolck, 2002).

### 1.3 Studies examining SRT in dyslexia

At present it is unclear whether individuals with dyslexia are impaired at the SRT tasks, as compared to typically developing (TD) control individuals. The key comparison in these studies is whether the difference in RTs between sequence and random blocks is significantly larger in TD individuals than in participants with dyslexia. Although a number of studies have indeed reported such findings (Jimenez-Fernandez, Vaquero, Jimenez, & Defior, 2011; Menghini, Hagberg, Caltagirone, Petrosini, & Vicari, 2006; Stoodley, Harrison, & Stein, 2006; Stoodley, Ray, Jack, & Stein, 2008; Vicari et al., 2005, 2003), others have not (Bussy et al., 2011; Deroost et al., 2010; Gabay, Schiff, & Vakil, 2012; Kelly, Griffiths, & Frith, 2002; Menghini et al., 2010; Rüsseler et al., 2006; Yang & Hong-Yan, 2011).

A number of explanations could account for the inconsistent pattern of findings. One possibility, of course, is that procedural learning impairments are in fact not present in dyslexia, contrary to the predictions of Nicolson and Fawcett (2007, 2011) and Ullman (2004). On this view, the pattern of results of impaired and unimpaired procedural learning in dyslexia is due to random chance, and a meta-analysis of SRT studies should not reveal a reliable impairment.

Second, procedural learning impairments might indeed be reliably present across individuals in the disorders, and the heterogeneity of results is due to noise or to insufficient power (e.g., small sample sizes) in some studies. On this perspective, a meta-analysis of SRT studies should reveal a deficit on this task, but no other variables (e.g., age, methodological factors) should account for any of the variability of findings across studies.

Third, procedural impairments may occur primarily in certain dyslexic subgroups, and/or under certain testing conditions. For example, it has previously been suggested that inconsistent findings pertaining to other cognitive and motor impairments in dyslexia might be due to deficits restricted largely to certain subgroups (e.g., Rosen, 2003; White et al., 2006). Additionally, the likelihood of compensation in the SRT task by declarative memory, which is primarily supported the medial temporal lobes, may change with age. Declarative memory supports learning, storage and retrieval of information (Squire, 1992; Squire, Stark, & Clark, 2004; Tulving & Markowitsch, 1998). Learning via the declarative memory system is occurs via binding arbitrarily related pieces of information together (Mayes, Montaldi, & Migo, 2007). Learning via the declarative memory system can be fast; learning can take place after a single exposure. However, with repeated exposures to the information the propensity learning takes places increases along with the efficiency stored information can be retrieved (Alvarez & Squire, 1994).

Research indicates declarative memory improves throughout childhood (Lum et al., 2010; Ofen et al., 2007; Ullman & Pierpont, 2005). In concert with behavioral data, the medial temporal have a comparable developmental trajectory, matuaring from childhood and into adolescence (Giedd et al., 1999; Ofen et al., 2007). Although the implicit version of the SRT task is designed to minimize the involvement of the declarative memory system, several studies show declarative memory and medial temporal involvement in participants who have neurodevelopmental or degenerative conditions affecting the basal ganglia and/or cerebellum (Beauchamp, Dagher, Panisset, & Doyon, 2008; Dagher, Owen, Boecker, & Brooks, 2001; Moody, Bookheimer, Vanek, & Knowlton, 2004; Rauch et al., 2007; Ullman & Pullman, 2013). In these groups, levels of implicit or perhaps even some explicit learning on the SRT task that are comparable to controls is achieved via activation of the medial temporal lobes. That is, declarative memory may be able to compensate for procedural memory impairments on the SRT task. These findings have form part of a larger literature suggesting that medial temporal lobe, underlies the learning of implicit as well as explicit knowledge (Chun, 2000; Poldrack & Rodriguez, 2003; Rose, Haider, Weiller, & Buchel, 2002; Ullman, 2008). The protracted developmental trajectory of declarative memory may mean compensation and subsequently smaller differences between dyslexic and control groups may be observed to a greater extent in samples comprising older children or adults. Thus the age of participants might account for differences in findings in the SRT task/dyslexia literature. Indeed, it is interesting to note that the age of participants in past research investing SRT task performance in dyslexia varies substantially ranging from a mean of less than 10 years to over 35 years (Jimenez-Fernandez et al., 2011; Menghini et al., 2006).

Variability in methodological conditions could also help explain learning variability in the SRT task in dyslexia. Multiple variants of this task have been employed in studies of dyslexia. The task varies in a number of respects. For instance, the length of training, for example, as measured by the number of exposures to the sequence (number of blocks times number of sequences per block), has varied substantially between studies, from as little as 10 (Stoodley et al., 2006) to as much as 108 (Deroost et al., 2010). Learning via the procedural memory system requires practice or repeated exposures to information (Packard & Knowlton, 2002). It could be that procedural learning takes place in individuals with dyslexia, however, more practice or exposure to information is required relative to age-matched controls. That is, individuals with dyslexia are ‘slow procedural learners’. Another possibility is that with extended training comes greater declarative memory-based compensation. Thus studies that provide more exposures to the sequence in SRT tasks might observe a smaller difference between dyslexic and control groups.

Also variable between studies is the length of the sequence used in the SRT task. In the dyslexia literature, this ranges from as few as five items (Vicari et al., 2003) to as many as 12 (Deroost et al., 2010; Rüsseler et al., 2006). Importantly, previous evidence suggests that shorter sequences are easier to learn than longer ones (Howard & Howard, 1989). Thus, differences between individuals with dyslexia and controls may only be observed for longer sequences.

Another potential methodological influence on study findings is the *type* of sequence. First order conditional (FOC) sequences and second order conditional (SOC) sequences have been used in past research into dyslexia (Deroost et al., 2010; Jimenez-Fernandez et al., 2011). In FOC sequences, the spatial location of the visual stimulus on the screen can be predicted from its preceding location. For example, if the visual stimulus appears in Position 1, there might be an 80% probability it will then appear in Position 2. In contrast, in SOC sequences the location of the visual stimulus cannot be predicted by its preceding location. That is, there is an equal probability between transitions from one spatial location to the next. Rather, the location that the visual stimulus will appear, can only predicted by multiple previous transitions.

A number of studies have shown implicit learning of SOC (and other higher order conditional) sequences involves the medial temporal lobes in addition to basal ganglia and cerebellum (Ergorul & Eichenbaum, 2006; Schendan, Searl, Melrose, & Stern, 2003). In accounting for these findings Poldrack and Rodriguez (2003) suggest the medial temporal lobes are necessary for representing information displaced over time or space, irrespective of whether learning or retrieval is implicit or explicit. In SOC sequences, since first order transitions occur with equal probability, learning can only take place if multiple preceding transitions are represented. This suggestion may explain why individuals with medial temporal lobe damage but intact basal ganglia and cerebellum can learn FOC sequences, but not SOC sequences (Curran, 1997). Also, fMRI studies investigating SRT task performance have shown medial temporal lobe activation when participants implicitly learning a SOC conditional sequence (Schendan et al., 2003) but not FOC conditional sequence (Thomas et al., 2004). Thus there is some evidence that implicit learning of SOC sequences may additionally be supported by the medial temporal lobe; a structure assumed to be intact in dyslexia (Hedenius, Ullman, Alm, Jennische, & Persson, 2013; Ullman & Pullman, 2013).

To understand whether SRT task performance is indeed at least partly explained by the above factors, they need to be systematically examined. In this report we first used meta-analysis to systematically synthesize the evidence related to performance of individuals with dyslexia on SRT tasks. The purpose of this analysis was to investigate whether individuals with dyslexia have poorer procedural learning compared to typically developing control participants who did not have dyslexia. We then used meta-regression to investigate whether there were systematic influences that might account for inconsistent findings in the literature examining SRT in dyslexia. Specifically, we investigated whether participants’ age and characteristics of the SRT task (number of exposures to the sequence, sequence length, and sequence type) could account for discrepancies between studies.

## 2 Method

### 2.1 Study design

We followed the methods used by previous meta-analyses of SRT task performance in other clinical groups, such as individuals with Parkinson's disease (Siegert et al., 2006; Siegert, Weatherall, & Bell, 2008). Articles were identified following searches in ERIC (hosted by EbscoHost), MEDLINE (hosted by OvidSP), EMBASE, CINAHL (hosted by EbscoHost), PsycInfo (hosted by EbscoHost) electronic databases to June 2013. The search strategy aimed to identify studies undertaken with samples comprising children or adults with developmental dyslexia who were administered a version of the SRT task. Details of all keywords and fields search are presented in Appendix A.

### 2.2 Study inclusion criteria

**Fig. 1:**
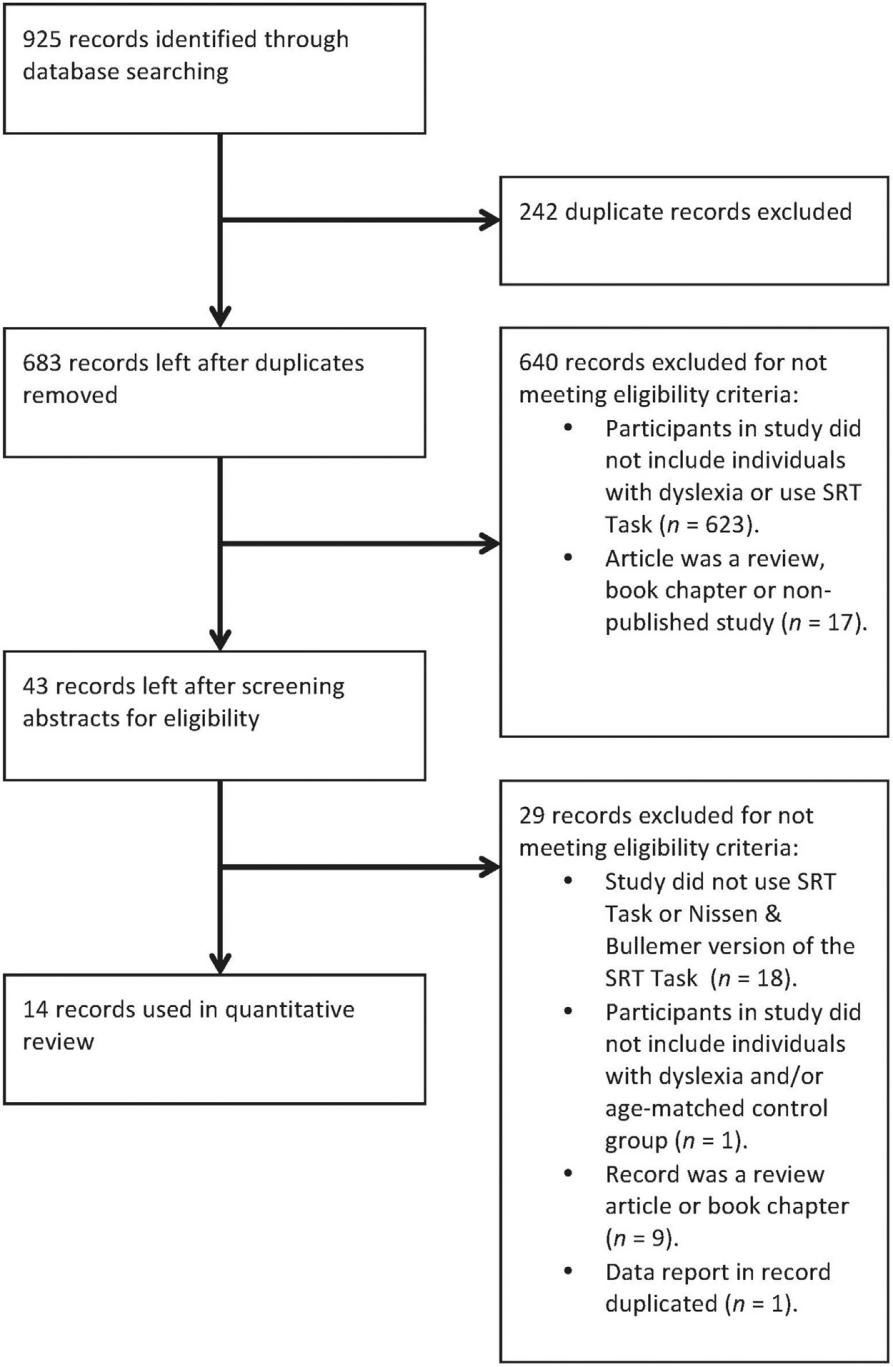
PRISMA flowchart showing selection of articles included in the meta-analysis.

The inclusionary criteria used in this meta-analysis were based on the protocols used by Siegert et al. (2006, 2008). First, since Nissen and Bullemer's (1987) original description of SRT task was first published in 1987, studies published before this date were excluded. Second, studies included in the meta-analysis were required to be published in a peer-review journal (written in any language) reporting on an original piece of research. Third, the study was required to have administered a version of Nissen and Bullemer's (1987) SRT task. That is, it was an implicit version of the task (no indication was given to the participant that there was any sort of sequence), and the structure of the task needed to involve presenting a series of blocks comprising sequenced spatial visual stimulus presentations that were followed by at least one block comprising random spatial visual stimulus presentations. Fourth, the study needed to have presented the SRT task to at least one group comprising individuals (children or adults) identified with developmental dyslexia (not alexia following adult-onset brain damage) and one control group comprising individuals of comparable age that did not have a developmental disorder (i.e., typically developing controls). These criteria served to identify studies with similar methodologies. Fig. 1 summarizes studies removed following application of each criterion according to PRISMA guidelines (Moher, Liberati, Tetzlaff, Altman, & The, 2009).

### 2.3 Study selection

After the removal of duplicate entries, one reviewer assessed all the abstracts. A random sample of 10% of all abstracts was assessed by a second reviewer. Any disagreements were resolved by discussion. Finally, the reviewers independently retrieved and screened full-text articles accordingly to the eligibility criteria. Inter-rater reliability was strong. For 42 out of the 43 articles (Cohen's kappa = .988) the reviewers independently agreed upon the suitability of each article for inclusion in the meta-analysis. Agreement about the suitability of one article was reached through consensus. A total of 14 published studies were included, and their data was extracted for the meta-analysis. A summary of each of study's participants and SRT task structure are summarized in Tables 1 and 2 respectively.

### 2.4 Effect size calculations and data extraction procedures

The standard method for comparing the performance of two groups on an SRT task tests whether the difference in RTs between the final random block and preceding block comprising sequenced stimulus presentations differs between the study and control group (e.g., Nissen & Bullemer, 1987). From each study, data was extracted to allow an effect size to be computed along with its variance that quantified this effect. A standardized mean difference (SMD) was used as the effect size measure. This metric describes differences between groups in standard deviation units. For this meta-analysis, the SMD was computed so that positive values indicated that the control group evidenced higher levels of procedural learning on the SRT task, as compared to the study group of individuals with dyslexia.

Following Siegert et al. (2006) the general formula to compute SMD for this interaction value is shown in Eq. (1) and variance in Eq. (2).

(1) $$\text{SMD}=\frac{{\bar{x}}_{\text{control}}-{\bar{x}}_{\text{dyslexic}}}{{\text{SD}}_{\text{pooled}}}$$

(2) $$\mathrm{var}(\text{SMD})=\frac{n_{\text{control}}+n_{\text{dyslexic}}}{n_{\text{control}}\times n_{\text{dyslexic}}}+\frac{{\text{SMD}}^{2}}{2(n_{\text{control}}+n_{\text{dyslexic}})}$$

where $\bar{x}$ is mean difference in RTs between the final random block and preceding sequence block (Table X showed blocks from each study used to compute the SMD).

SD_pooled_ is within-group standard deviation of the difference between the final random block and preceding block, pooled across the control and study group.

The result from each study included in the meta-analysis was described using a single effect size. For 11 studies, a single effect size was extracted for each study (Gabay et al., 2012; Jimenez-Fernandez et al., 2011; Kelly et al., 2002; Menghini et al., 2010, 2006; Rüsseler et al., 2006; Stoodley et al., 2006, 2008; Vicari et al., 2005, 2003; Yang, Bi, Long, & Tao, 2013). For three studies, it was necessary to average two sets of effect sizes reported. In the study by Bussy et al. (2011) effect sizes from analyses comparing two dyslexic subgroups to a control group were averaged to create a single effect size. In the Deroost et al. (2010) study, effect sizes were averaged from separate analyses that compared the dyslexic and control group on FOC and SOC sequence learning. In the study by Yang and Hong-Yan (2011) effect sizes were averaged from analyses that compared the dyslexic and control group performance on a SRT task which was completed by the left hand and then right hand. For six studies, it was necessary to impute a value for var(SMD) based on participants performance on other blocks of the SRT task (Deroost et al., 2010; Gabay et al., 2012; Jimenez-Fernandez et al., 2011; Kelly et al., 2002; Rüsseler et al., 2006; Yang & Hong-Yan, 2011). Comprehensive Meta-Analysis Software Package (Borenstein, Rothstein, & Cohen, 1999) was used to convert the extracted data to a common effect size and variance. Description of the data extracted from the studies is presented in Appendix B.

### 2.5 Meta-analytic procedures

To address the first question, that is, whether there was a difference between individuals with dyslexia and TD controls on sequence learning in SRT tasks, effect sizes were pooled and a weighted averaged effect size was computed using a random effects model (Hedges & Olkin, 1985). A significance test for the weighted average effect size was computed using an alpha level of .05. The use of a random effects model indicates we are assuming differences between study level effect sizes are the sum of sampling error (referred to as within-study variance) and ‘true’ effect size differences (referred to as between-study variance).

The second question addressed in this report was whether different methodological characteristics accounted for differences in study level effect sizes. In undertaking this analysis we first measured the total amount of heterogeneity using the *I*^2^ statistic (Higgins & Thompson, 2002). *I*^2^ describes the amount of heterogeneity in effect sizes (as a percentage or proportion) that is attributable to between-study effects (e.g., participant or methodological characteristics). Alternatively stated, the *I*^2^ statistic measures variability in effect sizes not attributable to sampling error. As a guideline Higgins, Thompson, Deeks, and Altman (2003) suggest that values of 25%, 50% and 75% correspond to low, moderate and high levels of heterogeneity respectively.

Finally, meta-regression (Greenland, 1987) was used to investigate whether participants’ age, sequence type, sequence length and number of exposures to the sequence accounted for variability in effect sizes.

## 3 Results

### 3.1 Evaluation of publication bias of included studies

**Fig. 2:**
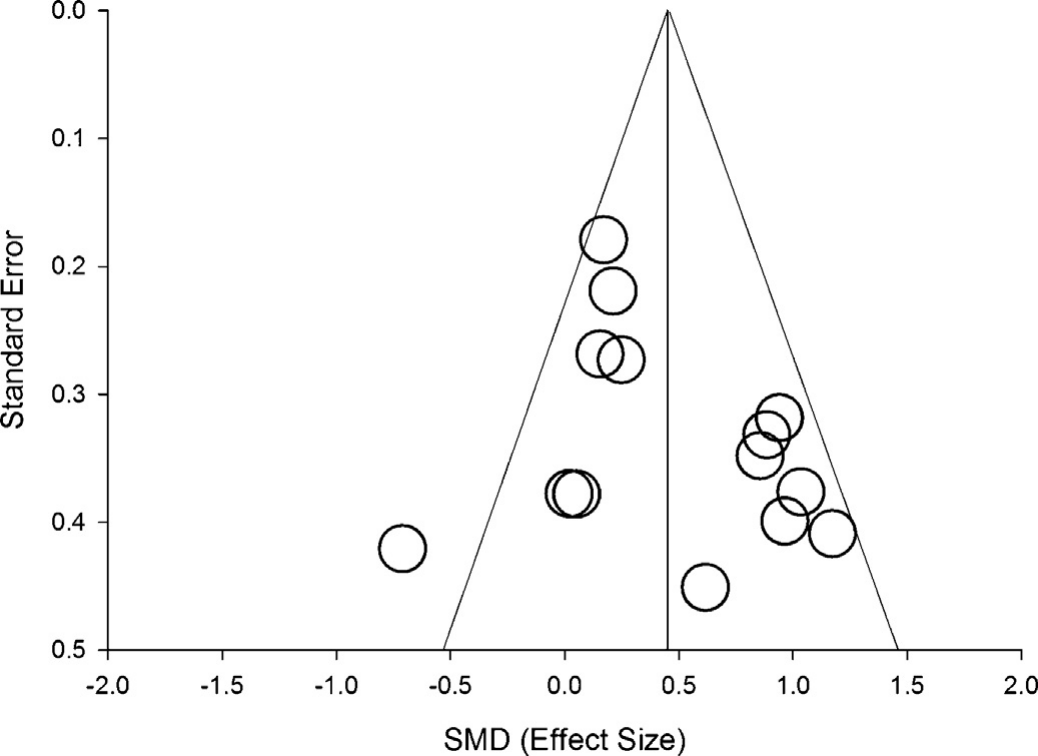
Funnel plot showing association between measure of precision (standard error) and effect size.

Preliminary analyses investigated the presence of publication bias using a funnel plot, which plots a measure of study precision (using standard error) against individual study effect sizes (Egger, Smith, Schneider, & Minder, 1997). These data are presented in Fig. 2. Using this approach, bias is considered to be present if effect sizes are asymmetrically distributed around the overall effect size when the study precision is low. When the study precision is high, there is less variability in study effect sizes. Egger's test of asymmetry was not found to be significant (Intercept = 1.793, *t* (12) = 1.32, *p* = .212). This suggests the distribution of effect sizes is adequately symmetrical and therefore that publication bias is unlikely.

### 3.2 Procedural learning in dyslexia

**Fig. 3:**
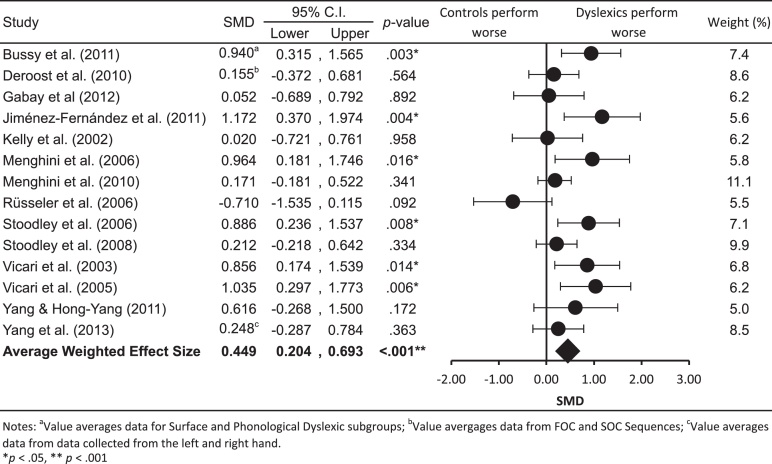
Forrest plot showing study level and average weighted effect sizes for individuals with dyslexia and control individual.

The first research question addressed whether or not individuals with dyslexia are worse at procedural learning than TD control individuals. The effect sizes computed for each study and the weighted average effect size are presented in Fig. 3. Positive SMD values indicate that the TD control group performed better than the dyslexia group, that is, that the control group showed more sequence learning as indicated by a larger RT difference between sequence and random blocks. The weighted average effect size was found to be .449 and highly significant (*p* < .001). This indicates that on average, individuals with dyslexia perform around half a standard deviation worse than controls of a comparable age on sequence learning in SRT tasks. According to Cohen's (1988) taxonomy this corresponds to a medium effect size.

Despite the finding that the overall effect size is statistically significant, inspection of Fig. 3 shows substantial variability in study level effect sizes. For example, the largest effect size observed was 1.172 and the smallest −.710 (which indicates that control individuals actually performed worse on the SRT task than individuals with dyslexia). Calculation of the *I*^2^ statistic indicated that 53.1% of variability between effect sizes represents true heterogeneity (i.e., differences between effect sizes not accounted for by sampling error). According to the guidelines by Higgins et al. (2003), this indicates that in this collection of studies there is moderate levels of heterogeneity. That is, there may be systematic influences that account for differences in study results.

### 3.3 What accounts for the heterogeneity in the findings?

Random-effects model meta-regression was used to investigate the second research question: whether the mean of age of participants in each study and methodological factors account for differences in study findings. The methodological factors examined were sequence type, sequence length and number of exposures to the sequence (see Table 2). In the study by Kelly et al. (2002) half the participants were tested on a sequence that was 9-elements long and the other half with an 8-element sequence. For the meta-regression analyses a value of 8.5 (the average of the two sequence lengths) was used to describe the sequence length in that study. Similarly, in that same study half of the participants were exposed to the sequence 96 times and the other half 84 times. The value used to describe the Number of Exposures to the Sequence in the meta-regression in that study was 90 (the average of the two values). Finally, for all studies Sequence Type was dummy coded so that FOC = 0 and SOC = 1.

To have sufficient statistical power in meta-regression an effect size to covariate ratio of 10:1 is suggested (see Borenstein, 2009). Since there were only 14 effect sizes (one from each study), separate meta-regressions were undertaken that tested one covariate at a time. In addition, we also investigated whether interactions between predictor variables accounted for the heterogeneity in effect sizes. The interaction term for continuous variables was created by centering and then multiplying variables. The interaction term using Sequence Type (which is a dichotomous variable) was created by multiplying this variable with other continuous variables. To preserve statistical power, only the interaction term was entered into the model. In these analyses the influence of the main effects were removed from the interaction term by regressing the interaction term on to the main effects and saving the standardized residuals using ordinary least squares regression. The residuals were then entered into the analysis as the interaction term. For example, to create the “Age × Sequence Length” interaction term, participants’ age and sequence length for each study were multiplied. The ensuing values were then regressed onto “Age” and “Sequence Length” and standardized residuals were saved and used as the covariate in the analysis.

The outcome variables used in the following meta-regressions were the effect sizes reported in Fig. 3. The exception was for analyses testing whether sequence type influenced effect sizes. As noted earlier, Deroost et al. (2010) tested participants on both FOC and SOC sequence types. To increase the number of data points for SOC sequences, only the effect size pertaining to the results for the SOC conditional sequences in that study was used in analyses investigating sequence type. Note that including both sets of results in the analyses would bias the results by treating dependent sets of results an independent (Tramèr, Reynolds, Moore, & McQuay, 1997). A summary of the results from the meta-regressions is presented in Table 3.

**Fig. 4:**
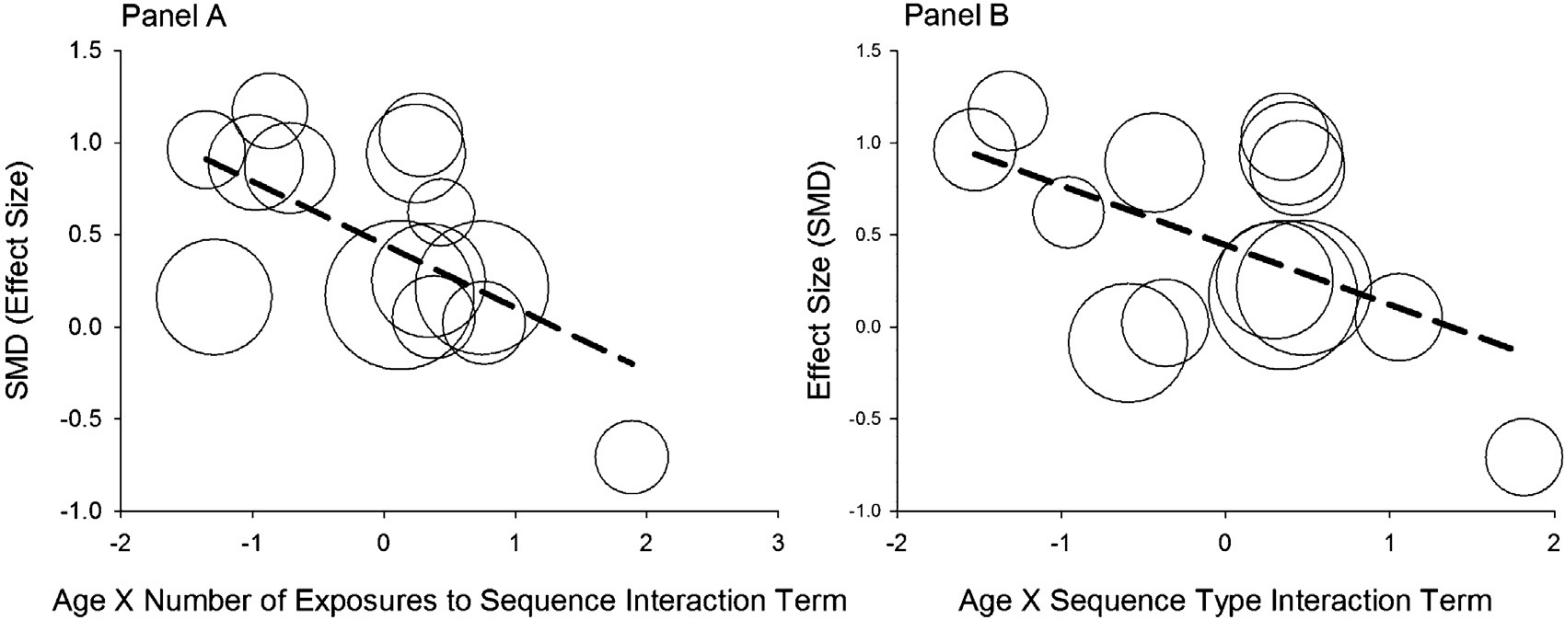
Scatterplot showing observed and predicted relationship between effect size and covariate. Covariate in Panel A is Age × Number Exposures to Sequence. Covariate in Panel B is Age × Sequence Type. Data points are proportionally sized according to their weight in the model.

Models 1–4 tested the contribution of age and methodological characteristics of the SRT task as main effects in accounting for differences in effect sizes; none were found to fit the data. However, two out of the six models testing an interaction were found to account for significant amounts of heterogeneity. Model 5 which tested the Age × Number of Exposures to the Sequence interaction term was found to be a significant predictor of effect sizes. This model accounted for 37% of between-study heterogeneity. This result indicates effect sizes become smaller in studies where participants are exposed to the sequence more times, but only when participants are older in age. In other words, the difference between the dyslexic and control groups on the SRT task decreases when more training to the sequence is provided and when participants are older. This model plotted against observed effect sizes is presented in Panel A in Fig. 4.

Model 6, which tested the Age × Sequence Type interaction term, was also found to be a significant predictor of effect sizes. This model accounted for 28% of between-study variance. This indicates that effect sizes become smaller for studies that presented SOC sequence, but only when participants were older. That is, the difference between dyslexic and control groups on the SRT task is smaller for SOC sequence than for FOC sequences, but only for older participants. Panel B in Fig. 4, plots this model against observed effect sizes.

## 4 Discussion

This report evaluated and synthesized available evidence of procedural learning deficits in dyslexia as indexed by sequence learning performance on SRT tasks. Following a systematic search of the literature, 14 studies were identified that were included in the meta-analysis. The weighted average effect size computed from these studies was found to be .449 (a medium effect size), which was statistically significant. This result indicates that on average, the mean difference in RTs between the final random block and preceding sequence block in SRT tasks is about half a standard deviation smaller in individuals with dyslexia than in typically developing control participants. The findings of this meta-analysis provide strong evidence in support of the hypothesis that procedural memory is impaired in dyslexia, and that this may help account for the reading deficits in the disorder (Nicolson & Fawcett, 2007; Ullman, 2004).

Using meta-regression, we also investigated potentially influencing factors that could account for the inconsistency of the findings in the SRT literature in dyslexia. We observed moderate to high levels of heterogeneity (which was formally quantified using the *I*^2^ statistic). As a reminder, in meta-analysis when using a random effects model to pool effect sizes, heterogeneity between effect sizes is assumed to reflect within-study and between-study variability. Within-study variability describes variability due to sampling error. Between-study variability refers to systematic influences on study effect size including participant or methodological characteristics. The observed *I*^2^ value of 53% indicates that 47% of heterogeneity between effect sizes reflected sampling error and 53% between-study error. Thus just over half of the observed heterogeneity in effect sizes in SRT studies in dyslexia appears to reflect systematic influences.

Two meta-regression models accounted for significant between-study heterogeneity. In one model, an interaction term comprising Age by Sequence Type was found to be a significant predictor of study level effect sizes (Model 6; see Table 2). This model accounted for 37% of variance in between-study heterogeneity. In the second model (Model 5; Table 2), the interaction term comprising Age by Number of Exposures to the Sequence was found to be a significant predictor of effect sizes. This model accounted for 28% of between-study heterogeneity. Collectively, these models show that the difference between dyslexic and control groups on SRT tasks becomes smaller as participants become older and also when a SOC sequence is used or, the sequence is presented more often.

An intriguing possibility we would like to emphasize relates to the significant ‘Age by Sequence Type’ interaction found. We suggest that this interaction might reflect compensatory mechanisms of the declarative memory system (Hedenius et al., 2013; Nicolson & Fawcett, 1990; Ullman & Pierpont, 2005; Ullman & Pullman, 2013). As discussed above, research suggests that the implicit learning of SOC sequences (and higher order sequences) additionally involves the medial temporal lobes and declarative memory (Curran, 1997; Ergorul & Eichenbaum, 2006; Schendan et al., 2003), which may be functioning relatively normally in dyslexia. But how about age as a factor in the interaction term? It is interesting to note that declarative memory improves throughout childhood and adolescence (for a review see Lum et al., 2010; Ullman, 2005). Thus, the capacity of the declarative memory system to compensate for procedural memory deficits may be more in older participants. Our suggestion would predict that, differences between individuals with dyslexia and control individuals would be greatest in early childhood and smallest in adulthood. But, importantly, under conditions where the SRT task is structured so as to place increasing demands on the medial temporal lobes such as using SOC sequence.

The interpretation of the meta-regression analysis showing a statistically significant ‘Age’ by ‘Number of Exposures to the Sequence’ interaction (see Table 2 Model 5) is less clear. As noted earlier, for this result, smaller effect sizes (or smaller differences between dyslexic and control groups) were predicted by studies with older participants and in SRT tasks that included more exposures to the sequence. One interpretation is that this finding might suggest the development of the procedural memory system is delayed in dyslexia. As a consequence, differences between individuals with dyslexia and controls on SRT tasks might be greatest when participants are young and there is limited opportunity to implicitly learn the sequence. Conversely, differences between groups might be minimal when participants are older and there are more exposures to the sequence. A second interpretation is that the significant interaction might be accounted for with respect to declarative memory based compensation. Increasing the number of exposures to the sequence might also provide greater opportunity for this memory system to play a role on the SRT task and given the developmental trajectory of this memory system, compensation is more likely to occur in older participants. Additional research is required to examine these possibilities further.

### 4.1 Limitations of meta-analysis and meta-regression

Two limitations need to be taken into account when interpreting the results presented in this report. First, results from the meta-analysis showing poorer procedural learning in dyslexia (see Fig. 2) cannot address the issue of causality between procedural learning and reading problems. All studies identified in the systematic search of the literature used a correlational research design. Therefore, the findings from our review should be interpreted to indicate that, at any particular single point in time, reading and procedural learning problems are evident in dyslexia. Second, interpreting results from meta-regression does require some caution (see Thompson & Higgins, 2002). This is because there might be measured or non-measured variables that correlate with the covariates, which in turn can lead to spurious claims about the relations between predictor and outcome variables. This is particularly important to note given the differences in participant characteristics noted in Table 1. For example, some studies ruled out the presence of ADHD in participants with dyslexia whereas others did not. The presence of ADHD or other comorbid disorder may also have an influence on effect sizes. It therefore needs to be noted that our discussion is speculative, necessarily tentative in nature and requires further investigation.

### 4.2 Clinical implications of meta-analyses findings

In the ongoing effort to improve detection rates and remediation of reading difficulties, the results from the current study suggest procedural memory is worthy of further consideration. With respect to detection, results presented in this report indicate poor procedural memory might be a risk factor for reading problems. Further research is needed to develop a practical, time-efficient assessment of procedural memory that can be used in clinical and other remediation contexts. The extent to which focusing on procedural memory in the context of remediation can lead to improved reading outcomes is crucially dependent on whether a causal link exists between poor procedural learning and reading problems; a topic for future research. The findings of these meta-analyses also raise issues regarding the focus for remediation-related research. Should remediation harness the compensatory potential of declarative memory, should intervention be directed to procedural memory, or both? More generally, the interactions observed between age and task performance on study findings, serve as a timely reminder that neurodevelopment needs to be taken into account to better understand the underlying nature of dyslexia (see Karmiloff-Smith, 1988).

## 5 Conclusion

In this report meta-analysis was used to evaluate the evidence for procedural learning deficits in individuals with dyslexia. The studies included in the meta-analyses measured procedural learning using SRT tasks. The weighted average effect size computed from 14 studies, representing data from 314 individuals with dyslexia and 317 control individuals, showed a significant difference between the groups in the SRT measure of procedural learning. We found that an interaction of age and methodological characteristics of the SRT task best accounted for differences between individual study findings. In sum, this report provides strong evidence of a procedural learning deficit in dyslexia. These reports also underline important areas for future research. In order to determine the clinical significance of impaired procedural memory studies are needed to examine potential causal links between this aspect of memory and reading difficulties.

## Figures and Tables

**Table:** 

Search strategy for CINAHL	
S1	(MH dyslexia+) OR (MH Reading Disorders+) OR (TI Dyslexia) OR (TI dyslexi*) OR (TI Reading) OR (TI read* N5 disorder*) OR (TI word N5 blind*) OR (TI text N5 blind*) OR (TI alexia) OR (TI read* N5 impair*) OR (TI read* N5 defic*) OR (TI read* N5 delay*) OR (TI read* N5 dysfunction*) OR (TI poor* N5 read*) OR (TI dysfluen* N5 read*) OR (TI slow* N5 read*) OR (TI remedial N5 read*) OR (TI word?blind*) OR (TI wordblind*) OR (TI read* N5 disorder*) OR (TI read* N5 disabilit*) OR (TI read* N5 problem*) OR (TI read* N5 difficulty*) OR (TI read* N5 deficien*) OR (TI read* N5 fluenc*) OR (TI read* N5 disab*) OR (AB Dyslexia) OR (AB dyslexi*) OR (AB Reading) OR (AB read* N5 disorder*) OR (AB word N5 blind*) OR (AB text N5 blind*) OR (AB alexia) OR (AB read* N5 impair*) OR (AB read* N5 defic*) OR (AB read* N5 delay*) OR (AB read* N5 dysfunction*) OR (AB poor* N5 read*) OR (AB dysfluen* N5 read*) OR (AB slow* N5 read*) OR (AB remedial N5 read*) OR (AB word?blind*) OR (AB wordblind*) OR (AB read* N5 disorder*) OR (AB read* N5 disabilit*) OR (AB read* N5 problem*) OR (AB read* N5 difficulty*) OR (AB read* N5 deficien*) OR (AB read* N5 fluenc*) OR (AB read* N5 disab*) OR (Dyslexia) OR (dyslexi*) OR (Reading) OR (read* N5 disorder*) OR (word N5 blind*) OR (text N5 blind*) OR (alexia) OR (read* N5 impair*) OR (read* N5 defic*) OR (read* N5 delay*) OR (read* N5 dysfunction*) OR (poor* N5 read*) OR (dysfluen* N5 read*) OR (slow* N5 read*) OR (remedial N5 read*) OR (word?blind*) OR (wordblind*) OR (read* N5 disorder*) OR (read* N5 disabilit*) OR (read* N5 problem*) OR (read* N5 difficulty*) OR (read* N5 deficien*) OR (read* N5 fluenc*) OR (read* N5 disab*)
S2	(TI serial reaction) OR (TI serial learn*) OR (TI sequence N5 learning) OR (TI implicit N5 sequence) OR (TI implicit learn*) OR (TI implicit N5 visuo?spatial) OR (TI implicit N5 visuospatial) OR (TI procedural learn*) OR (TI procedural mem*) OR (TI srt) OR (TI srtt) OR (TI motor skill learning) OR (AB serial reaction) OR (AB serial learn*) OR (AB sequence N5 learning) OR (AB implicit N5 sequence) OR (AB implicit learn*) OR (AB implicit N5 visuo?spatial) OR (AB implicit N5 visuospatial) OR (AB procedural learn*) OR (AB procedural mem*) OR (AB srt) OR (AB srtt) OR (AB motor skill learning) OR (serial reaction) OR (serial learn*) OR (sequence N5 learning) OR (implicit N5 sequence) OR (implicit learn*) OR (implicit N5 visuo?spatial) OR (implicit N5 visuospatial) OR (procedural learn*) OR (procedural mem*) OR (srt) OR (srtt) OR (motor skill learning)
S3	S1 AND S2
Search strategy for EMBASE	
S1	‘**dyslexia’**/exp OR **‘dyslexia’** OR **dyslexia**:ab,ti OR dyslexi*:ab,ti OR **reading**:ab,ti OR (**word** NEAR/5 blind*):ab,ti OR (**text** NEAR/5 blind*):ab,ti OR **alexia**:ab,ti OR (read* NEAR/5 impair*):ab,ti OR (read* NEAR/5 defic*):ab,ti OR (read* NEAR/5 delay*):ab,ti OR (read* NEAR/5 dysfunction*):ab,ti OR (poor* NEAR/5 read*):ab,ti OR (dysfluen* NEAR/5 read*):ab,ti OR (slow* NEAR/5 read*):ab,ti OR (**remedial** NEAR/5 read*):ab,ti OR ‘**word blindness**’:ab,ti OR ‘**word blind**’:ab,ti OR wordblind*:ab,ti OR (read* NEAR/5 disorder*):ab,ti OR (read* NEAR/5 disabilit*):ab,ti OR (read* NEAR/5 problem*):ab,ti OR (read* NEAR/5 difficulty*):ab,ti OR (read* NEAR/5 deficien*):ab,ti OR (read* NEAR/5 fluenc*):ab,ti OR (read* NEAR/5 disab*):ab,ti
S2	**‘procedural memory**’/exp OR ‘**procedural memory’** OR ‘**implicit memory**’/exp OR ‘**implicit memory’** OR (**serial** NEAR/5 **reaction**):ab,ti OR (**serial**:ab,ti AND learn*:ab,ti) OR (**sequence** NEAR/5 **learning**):ab,ti OR (**implicit** NEAR/5 **sequence**):ab,ti OR (**implicit**:ab,ti AND learn*:ab,ti) OR (**implicit** NEAR/5 ‘**visuo-spatial**’):ab,ti OR (**implicit** NEAR/5 **visuospatial**):ab,ti OR (**procedural**:ab,ti AND learn*:ab,ti) OR (**procedural**:ab,ti AND mem*:ab,ti) OR **srt**:ab,ti OR **srtt**:ab,ti OR (**motor**:ab,ti AND **skill**:ab,ti AND **learning**:ab,ti) OR (**serial**:ab,ti AND **reaction**:ab,ti AND **time**:ab,ti)
S3	S1 AND S2
Search strategy for ERIC	
S1	(DE dyslexia) OR (DE writing difficulties) OR (DE reading failure) OR (DE reading difficulties) OR (DE remedial reading) OR (TI Dyslexia) OR (TI dyslexi*) OR (TI Reading) OR (TI read* N5 disorder*) OR (TI word N5 blind*) OR (TI text N5 blind*) OR (TI alexia) OR (TI read* N5 impair*) OR (TI read* N5 defic*) OR (TI read* N5 delay*) OR (TI read* N5 dysfunction*) OR (TI poor* N5 read*) OR (TI dysfluen* N5 read*) OR (TI slow* N5 read*) OR (TI remedial N5 read*) OR (TI word?blind*) OR (TI wordblind*) OR (TI read* N5 disorder*) OR (TI read* N5 disabilit*) OR (TI read* N5 problem*) OR (TI read* N5 difficulty*) OR (TI read* N5 deficien*) OR (TI read* N5 fluenc*) OR (TI read* N5 disab*) OR (AB Dyslexia) OR (AB dyslexi*) OR (AB Reading) OR (AB read* N5 disorder*) OR (AB word N5 blind*) OR (AB text N5 blind*) OR (AB alexia) OR (AB read* N5 impair*) OR (AB read* N5 defic*) OR (AB read* N5 delay*) OR (AB read* N5 dysfunction*) OR (AB poor* N5 read*) OR (AB dysfluen* N5 read*) OR (AB slow* N5 read*) OR (AB remedial N5 read*) OR (AB word?blind*) OR (AB wordblind*) OR (AB read* N5 disorder*) OR (AB read* N5 disabilit*) OR (AB read* N5 problem*) OR (AB read* N5 difficulty*) OR (AB read* N5 deficien*) OR (AB read* N5 fluenc*) OR (AB read* N5 disab*) OR (SU Dyslexia) OR (SU dyslexi*) OR (SU Reading) OR (SU read* N5 disorder*) OR (SU word N5 blind*) OR (SU text N5 blind*) OR (SU alexia) OR (SU read* N5 impair*) OR (SU read* N5 defic*) OR (SU read* N5 delay*) OR (SU read* N5 dysfunction*) OR (SU poor* N5 read*) OR (SU dysfluen* N5 read*) OR (SU slow* N5 read*) OR (SU remedial N5 read*) OR (SU word?blind*) OR (SU wordblind*) OR (SU read* N5 disorder*) OR (SU read* N5 disabilit*) OR (SU read* N5 problem*) OR (SU read* N5 difficulty*) OR (SU read* N5 deficien*) OR (SU read* N5 fluenc*) OR (SU read* N5 disab*) OR (KW Dyslexia) OR (KW dyslexi*) OR (KW Reading) OR (KW read* N5 disorder*) OR (KW word N5 blind*) OR (KW text N5 blind*) OR (KW alexia) OR (KW read* N5 impair*) OR (KW read* N5 defic*) OR (KW read* N5 delay*) OR (KW read* N5 dysfunction*) OR (KW poor* N5 read*) OR (KW dysfluen* N5 read*) OR (KW slow* N5 read*) OR (KW remedial N5 read*) OR (KW word?blind*) OR (KW wordblind*) OR (KW read* N5 disorder*) OR (KW read* N5 disabilit*) OR (KW read* N5 problem*) OR (KW read* N5 difficulty*) OR (KW read* N5 deficien*) OR (KW read* N5 fluenc*) OR (KW read* N5 disab*)
S2	(TI serial reaction) OR (TI serial learn*) OR (TI sequence N5 learning) OR (TI implicit N5 sequence) OR (TI implicit learn*) OR (TI implicit N5 visuo?spatial) OR (TI implicit N5 visuospatial) OR (TI procedural learn*) OR (TI procedural mem*) OR (TI srt) OR (TI srtt) OR (TI motor skill learning) OR (AB serial reaction) OR (AB serial learn*) OR (AB sequence N5 learning) OR (AB implicit N5 sequence) OR (AB implicit learn*) OR (AB implicit N5 visuo?spatial) OR (AB implicit N5 visuospatial) OR (AB procedural learn*) OR (AB procedural mem*) OR (AB srt) OR (AB srtt) OR (AB motor skill learning) OR (SU serial reaction) OR (SU serial learn*) OR (SU sequence N5 learning) OR (SU implicit N5 sequence) OR (SU implicit learn*) OR (SU implicit N5 visuo? spatial) OR (SU implicit N5 visuospatial) OR (SU procedural learn*) OR (SU procedural mem*) OR (SU srt) OR (SU srtt) OR (SU motor skill learning) OR (KW serial reaction) OR (KW serial learn*) OR (KW sequence N5 learning) OR (KW implicit N5 sequence) OR (KW implicit learn*) OR (KW implicit N5 visuo?spatial) OR (KW implicit N5 visuospatial) OR (KW procedural learn*) OR (KW procedural mem*) OR (KW srt) OR (KW srtt) OR (KW motor skill learning)
S3	S1 AND S2
Search strategy for Medline	
S1	(Alexia or reading disabilities or Dyslexia or dyslexi* or Reading or (read* adj5 disorder*) or (word adj5 blind*) or (text adj5 blind*) or alexia or (read* adj5 impair*) or (read* adj5 defic*) or (read* adj5 delay*) or (read* adj5 dysfunction*) or (poor* adj5 read*) or (dysfluen* adj5 read*) or (slow* adj5 read*) or (remedial adj5 read*) or word#blind* or wordblind* or (read* adj5 disorder*) or (read* adj5 disabilit*) or (read* adj5 problem*) or (read* adj5 difficulty*) or (read* adj5 deficien*) or (read* adj5 fluenc*) or (read* adj5 disab*) or (read* adj5 impair*)).ti,ab,mp.
S2	(serial reaction or serial learn* or (sequence adj5 learning) or (implicit adj5 sequence) or implicit learn* or (implicit adj5 visuo#spatial) or (implicit adj5 visuospatial) or procedural learn* or procedural mem* or srt or srtt or motor skill learning or serial reaction time).ti,ab,mp.
S7	S1 AND S2
Search strategy for PsycInfo	
S1	(DE dyslexia) OR (DE Alexia) OR (DE reading disabilities) OR (TI Dyslexia) OR (TI dyslexi*) OR (TI Reading) OR (TI read* N5 disorder*) OR (TI word N5 blind*) OR (TI text N5 blind*) OR (TI alexia) OR (TI read* N5 impair*) OR (TI read* N5 defic*) OR (TI read* N5 delay*) OR (TI read* N5 dysfunction*) OR (TI poor* N5 read*) OR (TI dysfluen* N5 read*) OR (TI slow* N5 read*) OR (TI remedial N5 read*) OR (TI word?blind*) OR (TI wordblind*) OR (TI read* N5 disorder*) OR (TI read* N5 disabilit*) OR (TI read* N5 problem*) OR (TI read* N5 difficulty*) OR (TI read* N5 deficien*) OR (TI read* N5 fluenc*) OR (TI read* N5 disab*) OR (AB Dyslexia) OR (AB dyslexi*) OR (AB Reading) OR (AB read* N5 disorder*) OR (AB word N5 blind*) OR (AB text N5 blind*) OR (AB alexia) OR (AB read* N5 impair*) OR (AB read* N5 defic*) OR (AB read* N5 delay*) OR (AB read* N5 dysfunction*) OR (AB poor* N5 read*) OR (AB dysfluen* N5 read*) OR (AB slow* N5 read*) OR (AB remedial N5 read*) OR (AB word?blind*) OR (AB wordblind*) OR (AB read* N5 disorder*) OR (AB read* N5 disabilit*) OR (AB read* N5 problem*) OR (AB read* N5 difficulty*) OR (AB read* N5 deficien*) OR (AB read* N5 fluenc*) OR (AB read* N5 disab*) OR (SU Dyslexia) OR (SU dyslexi*) OR (SU Reading) OR (SU read* N5 disorder*) OR (SU word N5 blind*) OR (SU text N5 blind*) OR (SU alexia) OR (SU read* N5 impair*) OR (SU read* N5 defic*) OR (SU read* N5 delay*) OR (SU read* N5 dysfunction*) OR (SU poor* N5 read*) OR (SU dysfluen* N5 read*) OR (SU slow* N5 read*) OR (SU remedial N5 read*) OR (SU word?blind*) OR (SU wordblind*) OR (SU read* N5 disorder*) OR (SU read* N5 disabilit*) OR (SU read* N5 problem*) OR (SU read* N5 difficulty*) OR (SU read* N5 deficien*) OR (SU read* N5 fluenc*) OR (SU read* N5 disab*) OR (KW Dyslexia) OR (KW dyslexi*) OR (KW Reading) OR (KW read* N5 disorder*) OR (KW word N5 blind*) OR (KW text N5 blind*) OR (KW alexia) OR (KW read* N5 impair*) OR (KW read* N5 defic*) OR (KW read* N5 delay*) OR (KW read* N5 dysfunction*) OR (KW poor* N5 read*) OR (KW dysfluen* N5 read*) OR (KW slow* N5 read*) OR (remedial N5 read*) OR (KW word?blind*) OR (KW wordblind*) OR (KW read* N5 disorder*) OR (KW read* N5 disabilit*) OR (KW read* N5 problem*) OR (KW read* N5 difficulty*) OR (KW read* N5 deficien*) OR (KW read* N5 fluenc*) OR (KW read* N5 disab*)
S2	(DE Implicit Memory) OR (DE Implicit Learning) OR (TI serial reaction) OR (TI serial learn*) OR (TI sequence N5 learning) OR (TI implicit N5 sequence) OR (TI implicit learn*) OR (TI implicit N5 visuo?spatial) OR (TI implicit N5 visuospatial) OR (TI procedural learn*) OR (TI procedural mem*) OR (TI srt) OR (TI srtt) OR (TI motor skill learning) OR (AB serial reaction) OR (AB serial learn*) OR (AB sequence N5 learning) OR (AB implicit N5 sequence) OR (AB implicit learn*) OR (AB implicit N5 visuo?spatial) OR (AB implicit N5 visuospatial) OR (AB procedura learn*) OR (AB procedural mem*) OR (AB srt) OR (AB srtt) OR (AB motor skill learning) OR (SU serial reaction) OR (SU serial learn*) OR (SU sequence N5 learning) OR (SU implicit N5 sequence) OR (SU implicit learn*) OR (SU implicit N5 visuo?spatial) OR (SU implicit N5 spatial) OR (SU procedural learn*) OR (SU procedural mem*) OR (SU srt) OR (SU srtt) OR (SU motor skill learning) OR (KW serial reaction) OR (KW serial learn*) OR (KW sequence N5 learning) OR (KW implicit N5 sequence) OR (KW implicit learn*) OR (KW implicit N5 visuo?spatial) OR (KW implicit N5 visuospatial) OR (KW procedural learn*) OR (KW procedural mem*) OR (KW srt) OR (KW srtt) OR (KW motor skill learning)
S3	S1 AND S2

**Table:** 

Study	Description of data extracted from study
Bussy et al. (2011)	*F*-Value from repeated measures ANOVA's, undertaken separately each group, testing whether there was a significant in difference in RTs between Block 5 (final random block) and Block 4 (final sequence block). Also extracted were means from final sequence block (Block 4) and proceeding random block (Block 5). Means extracted from figure supplied by author.

Deroost et al. (2010)	Mean RTs for FOC and SOC sequences reported for each group from random block (Block 14) and preceding sequence block (Block 13). These data were extracted from Fig. 1. RTs standard deviations for Block 14 extracted from Fig. 2. RT *SD*'s for Block 13 estimated using data in Fig. 2 that reports a composite variable, which averages data from Blocks 13 and 15. To compute an effect size based on group *M's* and *SD's* requires an estimate of the correlation between random and sequence blocks. This value was obtained using data in the paper. Specifically, in addition to *SD's* for sequence and random blocks, the SD of the difference between Block 14 and composite sequence block comprising 13 & 15 was extracted from text.

Gabay et al. (2012)	Mean RTs from random block (Block 4) and preceding sequence block (Block 3) for each group. Data from Fig. 1. Also, results from ANOVA testing within-subject effect for difference between Block 3 and 4. These data were used to estimate standard deviation of the difference.

Jimenez-Fernandez et al. (2011)	Means RTs for each group's performance in the final random block (Block 10) and preceding sequence block (Block 9). These data were extracted from Fig. 3. *F*-Value from repeated measures ANOVA's that tested whether there was a significant difference in RTs between Block 10 and a composite variable comprising RT's from Block 9 and Block 11 (both sequenced blocks). Note the means and standard deviations from Blocks 9 and 11 were similar to Block 9. Thus, the composite appears to be an equivalent to Block 9.

Kelly et al. (2002)	Mean RTs from random block (Block 12) and preceding sequence block (Block 11) for each group. Data from Fig. 2. Also, results from ANOVA testing within-subject effect for difference between Block 3 and 4. These data used to estimate standard deviation of the difference.

Menghini et al. (2006)	*F*-value from an a 2 (Group: Dyslexic, Control) × 2 (Block: Block R2 (final random block), Block S5 (preceding Sequence Block) Factorial ANOVA.

Menghini et al. (2010)	*F*-value from an a 2 (Group: Dyslexic, Control) × 2 (Block: Block 6 (final random block), Block 5 (preceding Sequence Block) Factorial ANOVA.

Rüsseler et al. (2006)	Mean RTs for the random block (Block 10) and preceding sequence block (Block 9). These data were extracted from Fig. 2. Standard deviation for Block 10 was extracted from Fig. 3. Standard deviation for Block 9 was computed using data from a composite variable reported in the paper that averaged Block 9 & 11 (both sequenced blocks). Standard deviation data was extracted from Fig. 3. To compute an effect size for the interaction based on means and standard deviation for each group requires knowing the correlation between Block 9 and 10. This value was estimated using results from an ANOVA that tested whether there was a significant main effect for a difference between the composite variable comprising two sequenced blocks (Blocks 9 & 11) and the random block (Block 10). Additional information required to compute the correlation were the means and standard deviation for the composite variable (Blocks 9 & 11) and random variable (Block 10) collapsed across groups.

Stoodley et al. (2006)	Results from ANOVA testing significant interaction between group (Dyslexic, Control) × Block (Block 2 Sequence, Block 3, Random).

Stoodley et al. (2008)	Means and standard deviations RTs for second random block (Block 3) and preceding sequence block (Block 2). These data were from Table 2. The standard deviation of the difference was estimated using data comparing RTs for each group between sequence block (Block 1) and random Block (Block 2).

Vicari et al. (2003)	Means for final random block (Block 6) and preceding sequence block (Block 5). These data were reported in the text. Results from one-way repeated measures ANOVA, undertaken separately for each group, testing for significant difference in Block 6 (final random block) and Block 5 (preceding sequence block). These data was used to estimate the standard deviation of the change between blocks.

Vicari et al. (2005)	Mean RTs for final random block (Block 6) and preceding sequence block (Block 5). Data extracted from Fig. 1.
	Results (*p*-values) from repeated measures ANOVA (undertaken separately for control and dyslexic group testing whether a significant difference in RTs existed between Blocks 5 and 6. These data we used to estimate the standard deviation of the change between blocks.

Yang and Hong-Yan (2011)	Mean RTs for final random block (Block 4) and preceding sequence block (Block 3) for data reporting responses from left and right hand. Data extracted from Figs. 2 and 3. To compute standard deviation of the difference data and results from *t*-tests comparing within-group performance on Blocks 3 and 5 were extracted.

Yang et al. (2013)	Mean RTs for final random block (Block 5) and preceding sequence block (Block 4). Data reported in Table 2. Results (*t*-values) from paired samples *t*-tests testing whether a significant difference in RTs existed between Blocks 4 and 5 within each group. These data were used to estimate standard deviation of the difference between random and sequence blocks for both groups.

**Table 1:** Summary of study sample characteristics.

Study	Sample size		Mean age (years)		Gender		Presence of co-morbid developmental problems in dyslexic group	Additional matching characteristics of control group
	Dyslexic (*n*_study_)	Control (*n*_control_)	Dyslexic	Control	Dyslexic (% Female)	Controls (% Female)		
Bussy et al. (2011)	24^a^	18	11.2	10.6	c	c	ADHD ruled out	None reported.
Deroost et al. (2010)	28	28	13.5	13.6	60.7	60.7	ADHD ruled out	Education
Gabay et al. (2012)	14	14	25.6	25.1	57.1	78.6	ADHD ruled out	Non-verbal reasoning, socio-economic level.
Jimenez-Fernandez et al. (2011)	14	14	8.3	8.3	55.6	55.6	Not specified	Non-verbal reasoning
Kelly et al. (2002)	14	14	20.9	23.8	c	c	Not specified	Education and spatial reasoning skills.
Menghini et al. (2006)	14	14	42.1	37.2	71.4	71.4	ADHD ruled out	Education and handedness.
Menghini et al. (2010)	60	65	11.4	11.9	45	43.1	ADHD ruled out	Non-verbal intelligence.
Rüsseler et al. (2006)	12	12	28.8	32.8	75	58.3	No neurological diseases or problems	Handedness and performance IQ
Stoodley et al. (2006)	19	21	23.9	22.8	52.6	57.1	Not specified	Cognitive ability
Stoodley et al. (2008)	45	39^b^	10.1	9.4	c	c	Not specified	Non-verbal reasoning
Vicari et al. (2003)	18	18	10.5	10.2	33.3	38.9	Neurobehavioural problems ruled out	Intelligence and socio-economic level
Vicari et al. (2005)	16	16	11.6	11.4	25	31.3	Neurobehavioural problems ruled out	Socio-economic level
Yang and Hong-Yan (2011)	27	27	11.1	10.8	40.7	29.6	ADHD ruled out	Handedness and non-verbal reasoning
Yang et al. (2013)	9	12	12.63	12.24	33.3	33.3	ADHD ruled out	Handedness

a Comprises two dyslexic subgroups.

b A total of 44 control participants were included in the study, however, five were removed for the analyses involving the SRT task.

c Information not reported.

**Table 2:** Summary of study SRT task design.

Study	Sequence type	Sequence length	Blocks containing sequence	Blocks with random stimulus presentation	No. of trials per block	No. Exposures to sequence before final random block
Bussy et al. (2011)	FOC	10	2, 3, 4, 6	1, 5	90	27
Deroost et al. (2010)	Both	12	1–13, 15	14	100	108
Gabay et al. (2012)	SOC	8	1, 2, 3, 5	4	Blocks 1, 2, 3 = 160, 4 = 80, 5 = 80	60
Jimenez-Fernandez et al. (2011)	SOC	6	2^a^–9, 11	1, 2^a^, 10	Blocks 1 & 2 = 48, Blocks 3–10 = 60.	60
Kelly et al. (2002)	FOC	Half of participants presented with an 8-item sequence and half with 9-item sequence.	1–8, 10–11, 13–14, 16	9, 12, 15	76	Half of participants, 96 times & other half, 84 times.
Menghini et al. (2006)	FOC	9	2–6	1, 7	54	30
Menghini et al. (2010)	FOC	9	2–6	1, 7	54	30
Rüsseler et al. (2006)	SOC	12	2–9, 11	1, 10	120	80
Stoodley et al. (2006)	FOC	10	2	1, 3	100	10
Stoodley et al. (2008)	FOC	6	2	1, 3	Block 1 = 40, Block 2 = 84, Block 3 = 30.	14
Vicari et al. (2003)	FOC	5	2–5	1, 6	75	60
Vicari et al. (2005)	FOC	9	2–5	1, 6	54	24
Yang and Hong-Yan (2011)	SOC	6	2–3 & 5	1, 4	60	20
Yang et al. (2013)	FOC	8	2–4	1 & 5	48	18

a Block comprised random and sequence presentations.

**Table 3:** Results from meta-regression analyses investigating contribution of participant and SRT characteristics to differences in study level effect sizes. In meta-regression the *R*^2^ value describes the amount of true heterogeneity accounted for by the model. The *β* and *B* values describe the change in effect sizes following a one-unit change in the covariate. *β*-Values express the change in standard deviations and B values express the change in the original metric of the covariate. The *Q*_model_-statistic describes variability in effect sizes accounted by the model.

Model number/predictor in the model	*R*^2^	*Q*_Model_	*Q*_Residual_	*df*	*β*	*p*
Model 1: Age	0.04	0.637	14.098	1,12	0.43	.425
Model 2: Number of Exposures to Sequence	0.16	2.346	12.389	1,12	−0.40	.126
Model 3: Sequence Type	0.12	1.732	13.011	1,12	−0.34	.188
Model 4: Sequence Length	0.12	1.643	13.092	1,12	−0.33	.200
Model 5: Age × Number of Exposures to Sequence	0.37	5.414	9.321	1,12	−0.61	.020*
Model 6: Age × Sequence Type	0.28	4.127	10.615	1,12	−0.53	.042*
Model 7: Age × Sequence Length	0.06	0.820	13.916	1,12	−0.24	.365
Model 8: Sequence Type × Sequence Length	0.24	3.536	11.206	1,12	−0.49	.060
Model 9: Sequence Type × Number of Exposures to Sequence	0.04	0.575	14.168	1,12	−0.20	.449
Model 10: Sequence Length × Number of Exposures to Sequence	0.15	2.170	12.565	1,12	−0.38	.141

* *p* < .05.

## Appendix A Search syntax for databases

*Description of search syntax*: The search syntax is first identifies studies undertaken with participants who have dyslexia (designated as S1). This is achieved by searching for keywords in titles; abstracts MESH headings. The second step of the search (designated as S2) aims to identify studies that have investigated procedural learning (in any population). The third step of the search combines records identified in the S1 and S2 search using the ‘AND’ operator. This part of the search is designated as S3.
